# The “Perhaps” of Dental X-Ray

**Published:** 1922-07

**Authors:** Charles K. Field

**Affiliations:** Cincinnati, Ohio


### The "PERHAPS" of Dental X-Ray

BY CHARLES K. FIELD, D.M.D.
CINCINNATI, OHIO

### I.

A small rarified area at the apex of a tooth is—

1. Perhaps an abscess.
2. Perhaps a focal point of infection.
3. Perhaps the remains of an abscess which has been cured.
4. Perhaps (it is) the Mental Foramen.
5. Perhaps (it is) the Anterior Palatine Foramen.
6. Perhaps (it is) caused by the Drugs used in the treating of a dead tooth, whereby the lime salts have been dissolved.
7. Perhaps (it is) caused by a drop of Developer splashed on the film just before developing.
8. Perhaps (it is) caused by a little bubble of air on the film while it is immersed in the fixing solution.

### II.

In root canals which show incomplete root canal filling—

1. Perhaps the canal is so fine that there is no room for a gutta-percha point.
2. Perhaps the material used for root filling was non-resistant to the X-Ray.
3. Perhaps secondary dentin has filled the apical third.
4. Perhaps the canals have become entirely obliterated by old age.

### III.

With these few *Perhaps* in mind, *Perhaps* the dentist will be a little more careful how he diagnoses, *Perhaps*.

Groton Bldg., 7th & Race.

